# Clinical Psychology in Transition: Taking Responsibility and Broadening the Scope

**DOI:** 10.32872/cpe.13465

**Published:** 2023-12-22

**Authors:** Cornelia Weise, Winfried Rief

**Affiliations:** 1 Division of Clinical Psychology and Psychotherapy, Department of Psychology, Philipps-University of Marburg, Marburg, Germany

For many of us, December is the time to look back to what happened during the year. Very often we end up remembering all the challenges, difficulties, worries and burdens that have accompanied us throughout the year. This is also the case this year and not without a reason: The world is full of wars, there are crises and unstable political conditions in many countries around the globe, and not to forget the climate change that is speeding toward catastrophe (Lenton et al., 2023).

But… Should we really leave this Editorial’s review of the year at that? We don’t think so. Even if we have seen a lot of miserable things happen in 2023, there are also a lot of positive activities going on. Or in the words of Haruki Murakami:

“Where there is light, there must be shadow, and where there is shadow there must be light. There is no shadow without light and no light without shadow.” (Haruki Murakami, 1Q84)

In the ever-evolving landscape of clinical psychology, 2023 has witnessed remarkable strides that signal a paradigm shift in the discipline. This year has been marked by an expanded scope that addresses global challenges such as climate crises and wars, a heightened emphasis on patient and public involvement, and the establishment and fortification of crucial research initiatives. These positive developments not only signify the forward-thinking nature of clinical psychology but also underscore its relevance and adaptability in addressing contemporary societal issues.

**Addressing Global Challenges:** In response to the psychological impact of global challenges, the field of clinical psychology has expanded its purview. We are delighted to see that researchers in clinical psychology are taking responsibility and suggest ways how to improve mental health. In this issue, an Editorial by Pandi-Perumal and researchers from an impressive number of 22 different countries points to the consequences of war on mental health (Pandi-Perumal et al., 2023). The authors clearly express the need for international efforts to promote peace, humanitarian aid and mental health care. In an earlier issue, Asbrand, Michael, and colleagues (2023) discussed the impact of current challenges such as wars, societal polarization, and climate crisis on mental health in adolescents. The key recommendations of their paper include not only developing and expanding effective prevention and intervention programs, but also making a joint effort at various levels of society to enable effective changes. But also the activities of the field to overcome vaccination hesitancy to improve COVID-19 management indicated that clinical psychological concepts and intervention approaches are more and more considered relevant for tackling global challenges (Asbrand, Gerdes, et al., 2023; Bagarić & Jokić-Begić, 2022; Hysing et al., 2023; Lincoln & Rief, 2021; Wilson et al., 2022)

**Emphasizing Patient and Public Involvement**: The commitment to patient and public involvement in clinical psychology research is exemplified by initiatives mandated by renowned institutions such as the European Research Council (ERC) or the National Institute for Health Research (NIHR). For instance, the ERC, as a driving force in funding cutting-edge research across Europe, has been instrumental in promoting patient and public involvement as an integral component of research applications. Researchers seeking ERC grants are now required to demonstrate how they actively engage with patients and the public throughout the research process. Similarly, leading academic journals encourage researchers to engage with patients in the design, conduct, and dissemination of studies, recognizing the value of incorporating diverse perspectives to enhance the relevance and impact of medical and psychological research. These international initiatives not only elevate the standard of clinical psychology research but also align the discipline with a global ethos of inclusivity, ensuring that the voice of the patient and the public resonates in the development and implementation of mental health interventions.

**Establishing and Strengthening Research Initiatives:** This year has also seen the fortification of several research initiatives fostering mental health. These initiatives are characterized by their collaborative and cross-European nature, bringing together experts from diverse fields to tackle complex issues. Notable examples include projects focusing on the intersection of technology and mental health, initiatives aimed at reducing mental health disparities, and endeavors exploring the long-term impact of the global pandemic on mental well-being. The backbone of clinical psychology and psychiatry, namely the classification of mental disorders, is more and more challenged with suggestions for improvement or revision (see also our Special Issue “Innovations in ICD-11”, (Maercker, 2022a, 2022b) and the manuscript in this paper (Rief et al., 2023)). Intervention techniques and trainings in psychological treatments are searching for new frameworks, that help to overcome barriers of traditional psychotherapy theories (see our Special Issue on “Transtheoretical psychological treatments” that will be published early 2024). Our association, the European Association of Clinical Psychology and Psychological Treatment (EACLIPT) has emerged as a central force in promoting collaboration and advancing clinical psychology practices across Europe. Most importantly, the EACLIPT campaigns for better policies at a European level (e.g. by reporting to and building a direct exchange with members of the European Parliament). In addition, the series of EACLIPT webinars featuring renowned experts who present research on hot topics in clinical psychology and psychological treatment. In 2023, O’Connor talked about the psychology of suicide risk and Neuner focused on trauma treatment in refugees (Neuner, 2023; O’Connor, 2023).

As the year draws to a close, we are pleased to announce the completion of the **fifth volume** of Clinical Psychology in Europe. This milestone is a proof of the vibrant landscape of clinical psychology and would not have been possible without the collective support of our community.

Our heartfelt thanks go to our brilliant publisher, whose commitment to excellence and open science has been fundamental to our success. Our thanks also go to the reviewers for their careful evaluations and to the authors who entrust us with their groundbreaking work.

We look forward with enthusiasm to the forthcoming CPE volumes. Thank you for being an essential part of our journey.
